# Catheter-related bloodstream infections in patients receiving hemodialysis in a single Philippine tertiary-care center

**DOI:** 10.1017/ash.2023.283

**Published:** 2023-09-29

**Authors:** Dan Meynard Mantaring, Rohana Elise Rollan, Cybele Abad

## Abstract

**Background:** Information regarding catheter-related bloodstream infections (CRBSIs) among patients on hemodialysis in the Philippines is lacking. **Objective:** In this study, we described the clinical profile, CRBSI incidence density, and outcomes of patients in a single-center hemodialysis unit. **Methods:** A retrospective review of patients receiving hemodialysis (HD) through a central venous catheter (CVC) from January 2016 to December 2020 in a tertiary-care, private hospital was performed. Baseline demographic data were recorded, and CRBSI incidence density rates (no. of CRBSIs per 1,000 catheter days) were calculated. **Results:** Of 868 hemodialysis patients (57%), 499 used a CVC and were followed for 182,135 catheter days. Half were male (248 of 499, 49.7%) with a median age of 62 years (range, 24–90). Only 48 (9.6%) of 499 developed CRBSI, with an overall CRBSI incidence of 2.63 per 1,000 catheter days. Of those with CRBSI, 31 (64.6%) of 48 were female. The median age was 74.5 years (range, 30–90). Hypertension (40 of 48, 83.3%) and diabetes mellitus (26 of 48, 54.2%) were frequent comorbidities. Fever with chills was the most common symptom, occurring in 30 (62.5%) of 48 patients. Both gram-positive (n = 24) and gram-negative (n = 25) organisms were isolated. *Staphylococcus aureus* was the most common gram-positive isolate (14 of 25, 56%); isolates from the order Enterobacterales (12 of 24, 50%) were the most common gram-negative organisms. More CRBSIs occurred among those with a nontunneled versus tunneled CVCs (28 vs 20). The median time to CRBSI occurrence was 7 weeks (range, 0.43–280) from CVC insertion. The most common empiric treatment was either vancomycin (n = 28) or piperacillin-tazobactam (n = 26), which were also used in combination (11 of 28, 39.3%). Treatment involved CVC removal in most patients (34 of 48, 70.8%), either alone (n = 1), or with systemic antibiotic therapy (SAT; n = 16), or SAT plus antibiotic lock therapy (ALT; n = 17). The remainder (14 of 48, 29.2%) retained their CVCs because of difficult access, and received both SAT and ALT. Attributable mortality (6 of 9, 33%) and overall mortality (9 of 48, 18.5%) were high. Mortality of those whose CVC was retained was lower compared to those whose line was removed: (3 of 9, 33%) versus (6 of 9, 66%). **Conclusions:** The overall CRBSI rate in our hemodialysis unit was low and occurred more commonly in the older age group with a nontunneled CVC. Both gram-positive and gram-negative pathogens were common. CRBSI was associated with high attributable mortality. Successful treatment often required CVC, SAT, and ALT. However, CVC retention was a viable option in some patients with specific limiting factors such as difficult access.

**Figure:**
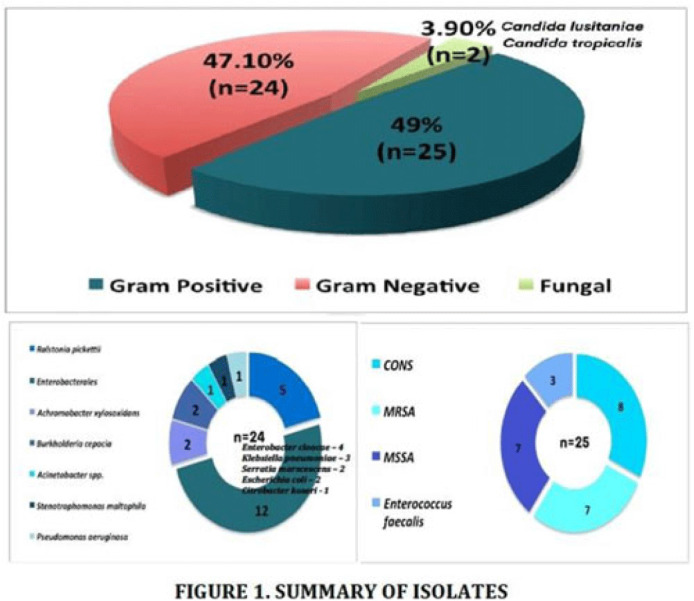

**Disclosures:** None

